# Supplementary material for: RUNX1 maintains the identity of the fetal ovary through an interplay with FOXL2
Source: Nat Commun. 2019 Nov 11;10:5116. doi: 10.1038/s41467-019-13060-1 (PMC6848188; doi:10.1038/s41467-019-13060-1)
Supplement: Supplementary file 1 — Supplementary Information [file 41467_2019_13060_MOESM1_ESM.pdf]

RUNX1 maintains the identity of the fetal ovary through an interplay with FOXL2

Nicol *et al.*

Supplementary Information

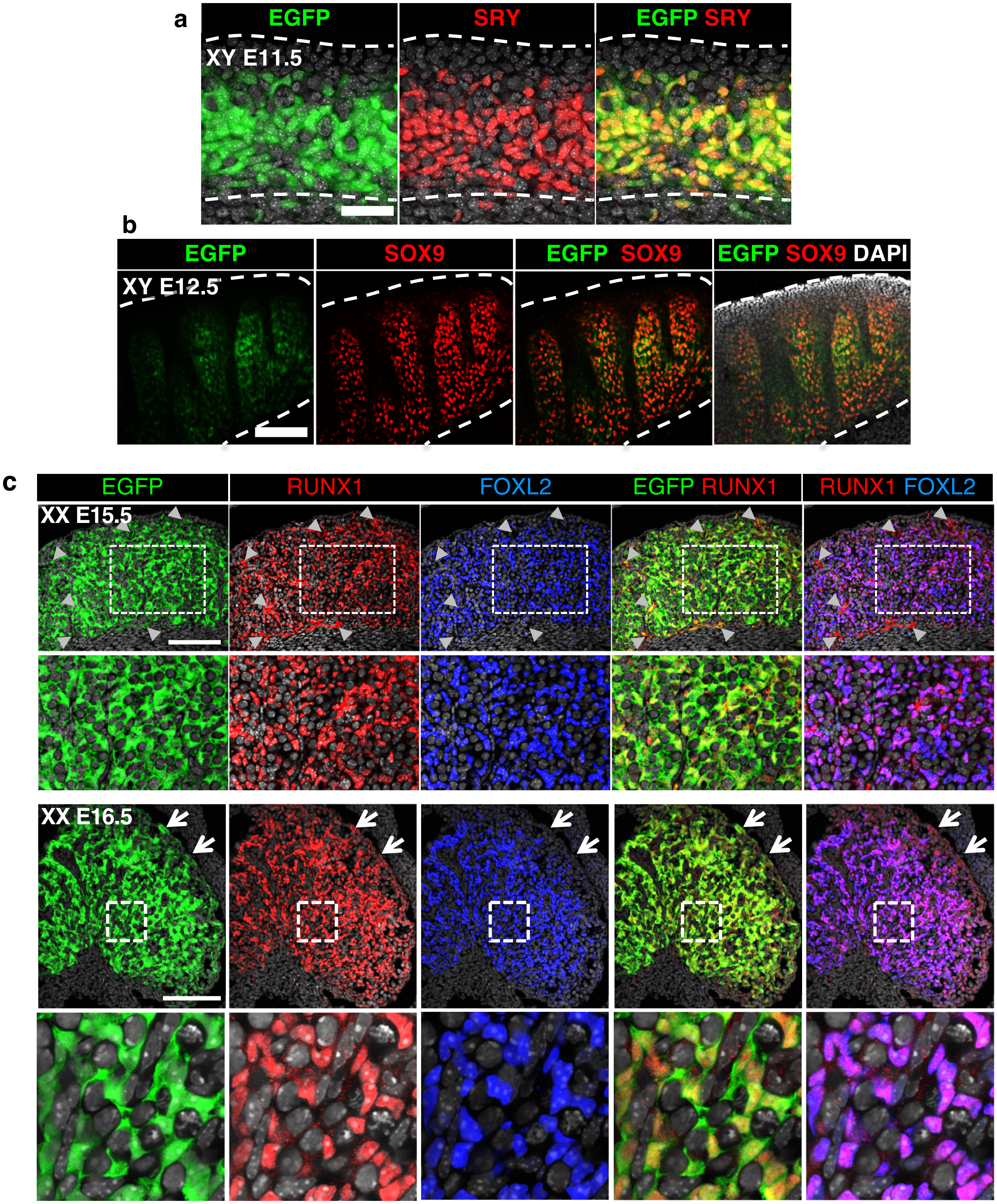

Supplementary Figure 1: *Runx1* expression in the fetal testis (a-b) and ovary (c). (a) Whole-mount immunofluorescence for SRY in XY Tg(Runx1-EGFP) gonads at E11.5. Scale bar: 50 μm. *Runx1*-EGFP is expressed exclusively in SRY+ supporting cells. **(b)** Whole-mount immunofluorescence for SOX9 in XY Tg(Runx1-EGFP) gonads at E12.5. Scale bar: 100 μm. Low levels of EGFP are detected in SOX9+ cells. (c) Immunofluorescence for EGFP, RUNX1 and FOXL2 on sections of XX Tg(Runx1-EGFP) gonads at E15.5 and E16.5. RUNX1 and FOXL2 are expressed in the same cells at E15.5. Note that some autofluorescence in green and red channels is found in the blood vessels at E15.5 (arrowheads). At E16.5, some GFP+/RUNX1+/FOXL2- cells appear at the cortex (arrows). Scale bars: 100 μm. At least 3 independent biological replicates were analyzed, and the images presented in the figures are representative of all replicates.

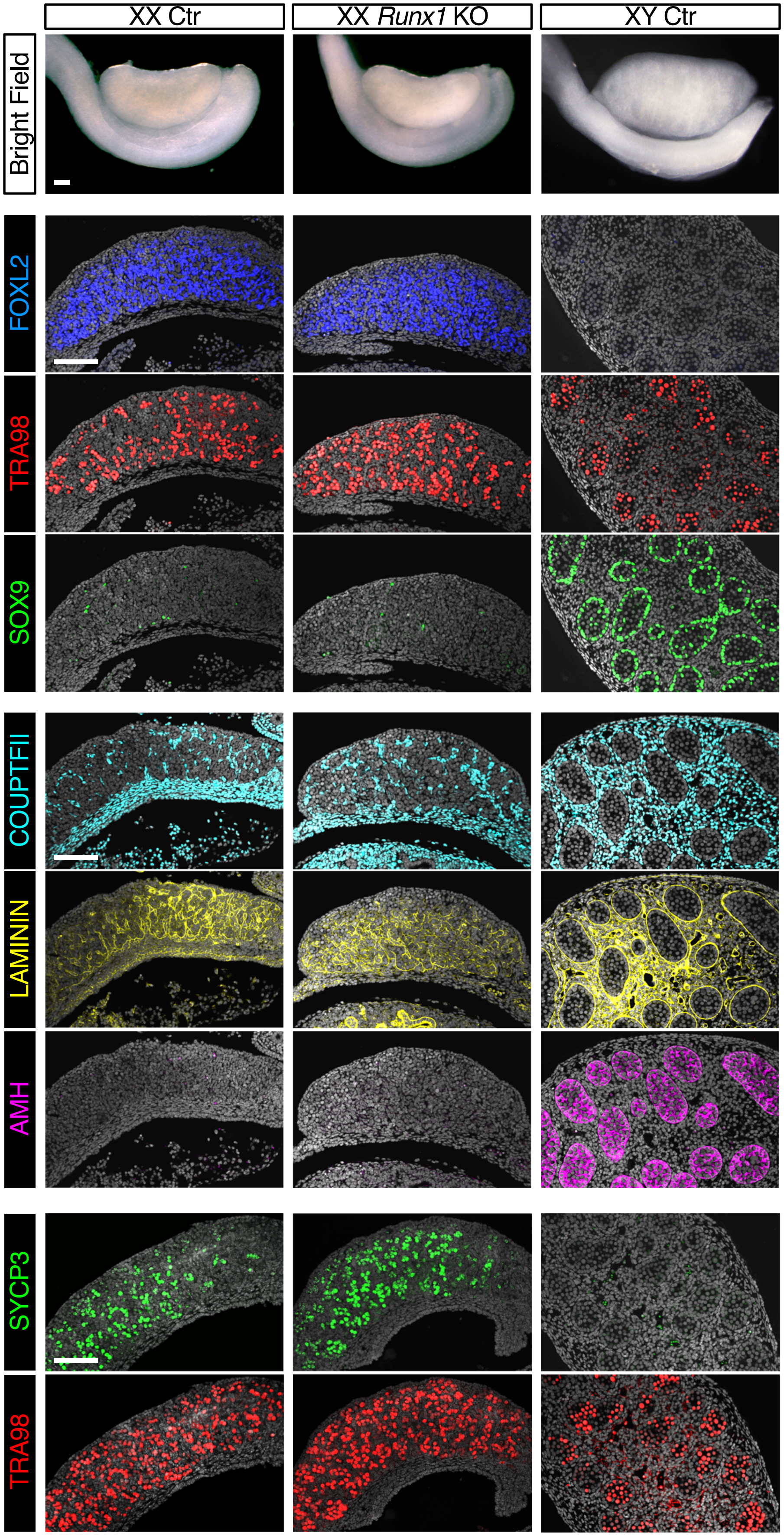

Supplementary Figure 2: Bright field images of E14.5 gonads and single channel immunofluorescences images for E14.5 XX gonads shown in Fig. 4 a-c. Scale bars: 100 μm. At least 3 independent biological replicates were analyzed, and the images presented in the figures are representative of all replicates.

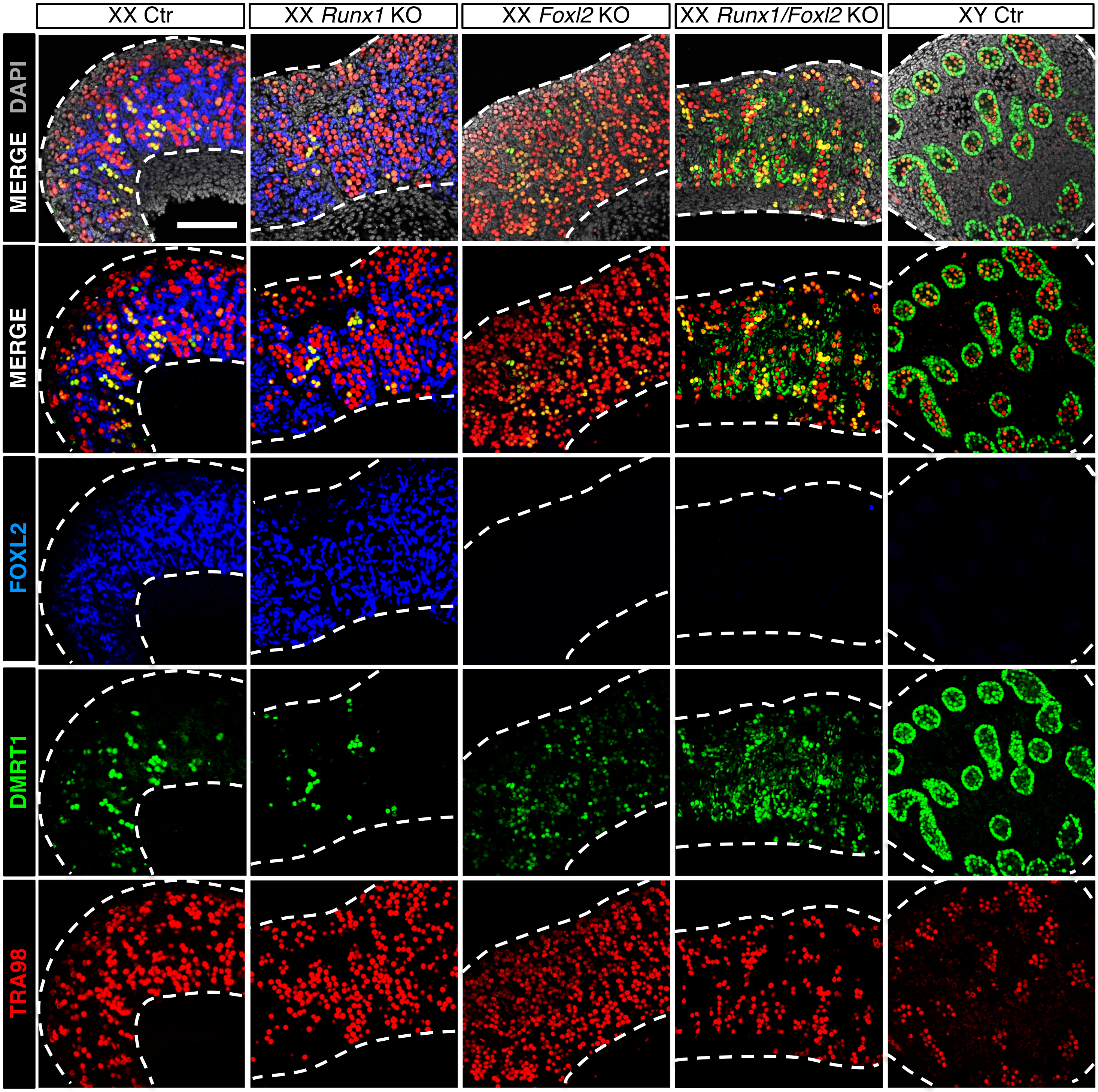

Supplementary Figure 3: Single channels for the FOXL2/DMRT1/TRA98 immunofluorescences at E15.5 shown in Fig. 5 a-e. Scale bar: 100 μm. Dotted lines outline the gonads. At least 3 independent biological replicates were analyzed, and the images presented in the figures are representative of all replicates.

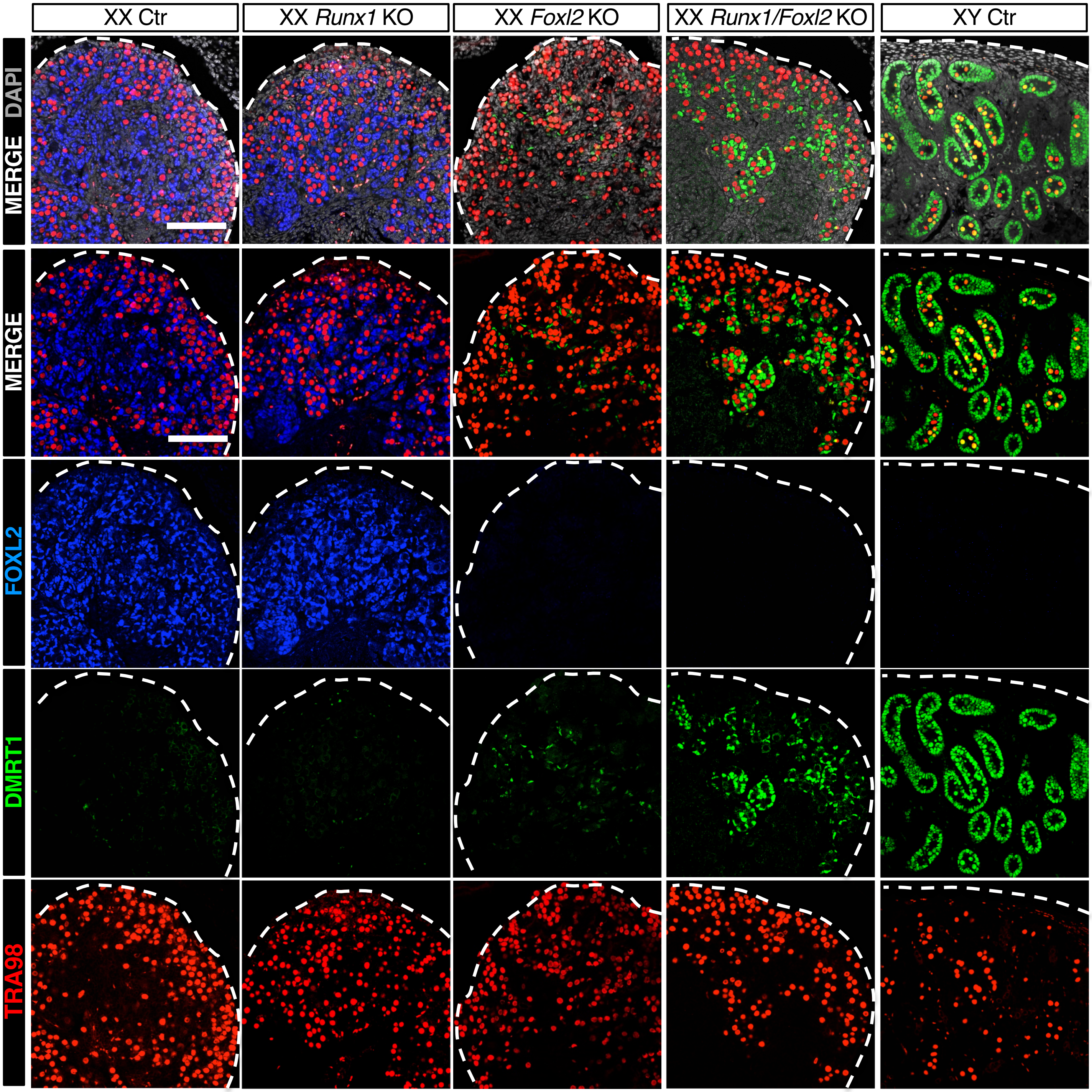

Supplementary Figure 4: Single channels for the FOXL2/DMRT1/TRA98 immunofluorescence at birth shown in Fig. 5 f-j. Scale bar: 100 μm. Dotted lines outline the gonads. At least 3 independent biological replicates were analyzed, and the images presented in the figures are representative of all replicates.

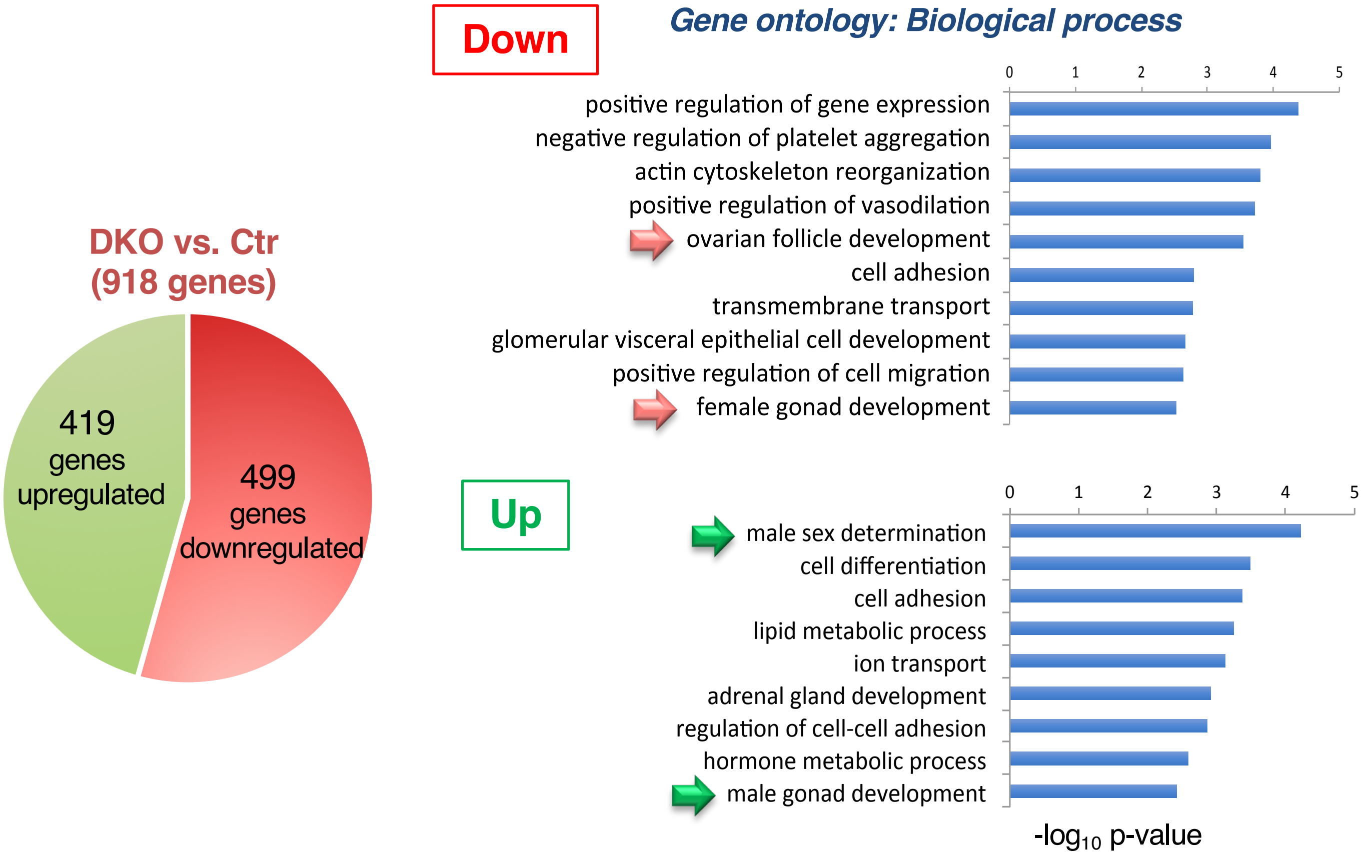

Supplementary Figure 5: Gene ontology biological process analysis of genes downregulated or upregulated in *Runx1/Foxl2* DKO vs. control ovaries at birth. Top GO-terms are shown. Analysis performed using DAVID 6.8 on genes significantly changed with a fold-change >1.5 and $P<0.05$, one-way ANOVA.

Supplementary Table 1: List of antibodies used for immunofluorescence in this study.

Antibodies used for immunofluorescence			
Protein	***Concentration***	***Species***	***Origin***
EGFP	1:500	Chicken	ab13970 ; Abcam
PECAM-1 (CD31)	1:1000	Rat	#550274; BD
SRY	1:200	Rabbit	*gift from D. Wilhelm, Monash University, Australia*
SF1	1:500	Rabbit	*gift from K. Morohashi, Kyushu University, Japan*
FOXL2	1:300	Goat	NB100-1277; NOVUS
RUNX1	1:300	Rabbit	*gift from S. Brenner-Morton and T. Jessell (Columbia University, USA)*
SOX9	1:500	Rabbit	*gift from K. Morohashi, Kyushu University, Japan*
TRA98	1:1000	Rat	RK-73-003, MBL International
COUP-TFII	1:300	Mouse	PP-H7147-10; R&D Systems
LAMININ	1:500	Rabbit	L9393; Sigma
AMH	1:500	Goat	Santa Cruz
SYCP3	1:300	Rabbit	ab15093 ; Abcam
DMRT1	1:300	Rabbit	*gift from D. Zarkower, University of Minnesota*

Supplementary Table 2: List of Taqman probes used for qPCR in this study

Taqman probes used for real time PCR analyses	
Gene	***Taqman probe***
Amh	Mm01172799_g1
Cyp19a1	Mm00484049_m1
Dhh	Mm01310203_m1
Dmrt1	Mm00443809_m1
Fgf9	Mm00442795_m1
Foxl2	Mm00843544_m1
Gapdh	Mm99999915_g1
Nr5a1 (Sf1)	Mm00446826_m1
Sox9	Mm00448840_m1

Supplementary Table 3: List of primers used for qPCR in this study

Primers used for real time PCR analyses		
Gene	***Forward Primer***	***Reverse Primer***
Foxp1	GGTCTGAGACAAAAAGTAACGGA	CGCACTCTAGTAAGTGGTTGC
Fst	TGCTGCTACTCTGCCAGTTC	GTGCTGCAACACTCTTCCTTG
Gapdh	TTCACCACCATGGAGAAGGC	GGCATGGACTGTGGTCATGA
Itpr2	CCTCGCCTACCACATCACC	TCACCACTCTCACTATGTCGT
Mvh	GAGAACACATCTACAACTGGTGG	AAACCTCTGTTTCCAAAGCCC
Pdgfc	GCCAAAGAACGGGGACTCG	AGTGACAACTCTCTCATGCCG
Pla2r1	CAGGGCATCGCCCAAGATTT	TTCAGGCTCTCGCTCTGGAT
Runx1 (Trout)	CTGTCAGAACACCCTGGAGAACT	GTACCGTCAGGGATATCACCAAG
Runx1 (Goat)	GCAGGCAATGATGAAAACTACT	TAGGTGGCGACTTGCGGTG
Runx1	GCAGGCAACGATGAAAACTACT	GCAACTTGTGGCGGATTTGTA
Runx2	ATCCCCATCCATCCACTCCA	GCCAGAGGCAGAAGTCAGAG
Runx3	CAGGTTCAACGACCTTCGAT	CTTGGGTAGGGTTGGTGAACA
Ryr2	ACGGCGACCATCCACAAAG	AAAGTCTGTTGCCAAATCCTTCT
Wnt4	AGACGTGCGAGAAACTCAAAG	GGAACTGGTATTGGCACTCCT
