# Supplementary material for: RUNX1 maintains the identity of the fetal ovary through an interplay with FOXL2
Source: Nat Commun. 2019 Nov 11;10:5116. doi: 10.1038/s41467-019-13060-1 (PMC6848188; doi:10.1038/s41467-019-13060-1)
Supplement: Supplementary file 3 — Reporting Summary [file 41467_2019_13060_MOESM3_ESM.pdf]

nature research

Corresponding author(s): Humphrey Yao

Last updated by author(s): Jul 17, 2019

Reporting Summary

Nature Research wishes to improve the reproducibility of the work that we publish. This form provides structure for consistency and transparency in reporting. For further information on Nature Research policies, see Authors & Referees and the Editorial Policy Checklist.

Statistics

For all statistical analyses, confirm that the following items are present in the figure legend, table legend, main text, or Methods section.

n/a	Confirmed	
☐	☒	The exact sample size (*n*) for each experimental group/condition, given as a discrete number and unit of measurement
☐	☒	A statement on whether measurements were taken from distinct samples or whether the same sample was measured repeatedly
☐	☒	The statistical test(s) used AND whether they are one- or two-sided<br>*Only common tests should be described solely by name; describe more complex techniques in the Methods section.*
☒	☐	A description of all covariates tested
☐	☒	A description of any assumptions or corrections, such as tests of normality and adjustment for multiple comparisons
☐	☒	A full description of the statistical parameters including central tendency (e.g. means) or other basic estimates (e.g. regression coefficient) AND variation (e.g. standard deviation) or associated estimates of uncertainty (e.g. confidence intervals)
☒	☐	For null hypothesis testing, the test statistic (e.g. *F*, *t*, *r*) with confidence intervals, effect sizes, degrees of freedom and *P* value noted<br>*Give P values as exact values whenever suitable.*
☒	☐	For Bayesian analysis, information on the choice of priors and Markov chain Monte Carlo settings
☒	☐	For hierarchical and complex designs, identification of the appropriate level for tests and full reporting of outcomes
☒	☐	Estimates of effect sizes (e.g. Cohen's *d*, Pearson's *r*), indicating how they were calculated

Our web collection on statistics for biologists contains articles on many of the points above.

Software and code

Policy information about availability of computer code

Data collection

Microarray data was obtained using the GeneChip® Command Console Software (AGCC; Version 3.2) and Expression Console (Version 1.2).

Data analysis

For the microarray:
Gene expression analyses were conducted with Partek software (St. Louis, Missouri) using a one-way ANOVA comparing the RMA normalized log2 intensities.

For the ChIP-seq:
ChIP-seq libraries were sequenced as single-end 75-mers by Illumina NextSeq 500, then filtered to retain only reads with average base quality score >20. Reads were mapped against the mouse mm10 reference genome using Bowtie v1.2 with parameter "-m 1" to collect only uniquely-mapped hits. Duplicate mapped reads were removed using Picard tools MarkDuplicates.jar (v1.110). After merging the replicate datasets, binding regions were identified by peak calling using HOMER v.4.9 67 with FDR<1e-5. Called peaks were subsequently re-defined as 300mers centered on the called peak midpoints and filtered for a 4-fold enrichment over input and over local signal. Genomic distribution of RUNX1-bound regions was determined based on Refseq gene models as downloaded from the UCSC Genome Browser as of August 09, 2017. Enriched motifs were identified using HOMER findMotifsGenome.pl de novo motif analysis with parameter "-size given". For RUNX1 and FOXL2 ChIP-seq comparisons, binding peaks that had at least 1 bp in common were considered overlapping. Peaks were assigned to the nearest gene based on RefSeq.

For manuscripts utilizing custom algorithms or software that are central to the research but not yet described in published literature, software must be made available to editors/reviewers. We strongly encourage code deposition in a community repository (e.g. GitHub). See the Nature Research guidelines for submitting code & software for further information.

Data

Policy information about availability of data

All manuscripts must include a data availability statement. This statement should provide the following information, where applicable:

- Accession codes, unique identifiers, or web links for publicly available datasets
- A list of figures that have associated raw data
- A description of any restrictions on data availability

Microarray and ChIP-seq data generated in this study have been deposited in GEO under accession numbers GSE129038 and GSE128767 respectively. The ChIP-seq data are available in the ReproGenomics Viewer (https://rgv.genouest.org).
Raw data underlying all reported mean values in graphs are provided in the Source Data File. All other relevant data supporting the key findings of this study are available in the supplementary files.

Field-specific reporting

Please select the one below that is the best fit for your research. If you are not sure, read the appropriate sections before making your selection.

☒ Life sciences ☐ Behavioural & social sciences ☐ Ecological, evolutionary & environmental sciences

For a reference copy of the document with all sections, see nature.com/documents/nr-reporting-summary-flat.pdf

Life sciences study design

All studies must disclose on these points even when the disclosure is negative.

Sample size

All experiments using mouse samples were performed on at least 3 biological replicates.

Data exclusions

No data were excluded from the analyses.

Replication

Microarray data were confirmed by qPCR to verify the reproducibility. Immunofluorescence experiments were replicated several times using independant biological replicates and attempts at replication of the results were successful.

Randomization

samples were allocated into experimental group by genotype.

Blinding

Blinding was not performed in our study

Reporting for specific materials, systems and methods

We require information from authors about some types of materials, experimental systems and methods used in many studies. Here, indicate whether each material, system or method listed is relevant to your study. If you are not sure if a list item applies to your research, read the appropriate section before selecting a response.

Materials & experimental systems

n/a	Involved in the study	
☐	☒	Antibodies
☒	☐	Eukaryotic cell lines
☒	☐	Palaeontology
☐	☒	Animals and other organisms
☒	☐	Human research participants
☒	☐	Clinical data

Methods

n/a	Involved in the study	
☐	☒	ChIP-seq
☒	☐	Flow cytometry
☒	☐	MRI-based neuroimaging

Antibodies

Antibodies used

EGFP 1:500 Chicken ab13970 ; Abcam
PECAM-1 (CD31) 1:1000 Rat #550274; BD
SRY 1:200 Rabbit gift from D. Wilhelm, Monash University, Australia
SF1 1:500 Rabbit gift from K. Morohashi, Kyushu University, Japan
FOXL2 1:300 Goat NB100-1277; NOVUS
SOX9 1:500 Rabbit gift from K. Morohashi, Kyushu University, Japan
TRA98 1:1000 Rat RK-73-003, MBL International
COUP-TFII 1:300 Mouse PP-H7147-10; R&D Systems
LAMININ 1:500 Rabbit L9393; Sigma
AMH 1:500 Goat Santa Cruz

SYCP3 1:300 Rabbit ab15093 ; Abcam
DMRT1 1:300 Rabbit gift from D. Zarkower, University of Minnesota

Validation

Antibody validation is based on the statement of manufacturers for the commercial antibodies, and based on previously published data for the others (see Nicol et al., 2018 and others)

Animals and other organisms

Policy information about studies involving animals; ARRIVE guidelines recommended for reporting animal research

Laboratory animals

Tg(Runx1-EGFP) reporter mouse was purchased from MMRRC (MMRRC_010771-UCD)
Male and females embryos were collected at ages E11.5, E12.5, E14.5, E15.5, E16.5 and birth.

CD-1 mice were purchased from Charles River (stock number 022)
Male and females embryos were collected at ages E11.5, E12.5, E13.5, E14.5, E16.5, E18.5 and postnatal day 3.

Runx1+/- (B6.129S-Runx1tm1Spe/J) and Runx1f/f (B6.129P2-Runx1tm1Tani/J) mice were purchased from the Jackson Laboratory (stock numbers 005669 and 008772, respectively)
Sf1-CreTg/Tg mice34 (B6D2-Tg(Nr5a1-cre)2Klp) were provided by late Dr. Keith Parker
Foxl2+/- mice61 (B6;129-Foxl2<tm1Gpil>) were provided by Dr. David Schlessinger (National Institute on Aging).
Runx1 KO mice (Sf1Cre+/Tg; Runx1f/-) were generated by crossing Runx1f/f females with Sf1-Cre+/Tg; Runx1+/- males. Controls were Sf1-Cre+/+; Runx1+/f littermates. Female were collected at E14.5 and P0.

Runx1/Foxl2 double knockout mice (Sf1Cre+/Tg; Runx1f/-; Foxl2-/-) were generated by crossing Runx1f/f; Foxl2+/- females with Sf1Cre+/Tg; Runx1+/-; Foxl2+/- males. This cross also generated the single knockouts for Runx1 (Sf1Cre+/Tg; Runx1f/-; Foxl2+/+) and Foxl2 (Sf1Cre+/+; Runx1f/+; Foxl2-/-), and control littermates (Sf1-Cre+/+; Runx1+/f; Foxl2+/+).
Male and females were collected at E15.5 and P0

Wild animals

Provide details on animals observed in or captured in the field; report species, sex and age where possible. Describe how animals were caught and transported and what happened to captive animals after the study (if killed, explain why and describe method; if released, say where and when) OR state that the study did not involve wild animals.

Field-collected samples

For laboratory work with field-collected samples, describe all relevant parameters such as housing, maintenance, temperature, photoperiod and end-of-experiment protocol OR state that the study did not involve samples collected from the field.

Ethics oversight

All mouse procedures were approved by the National Institutes of Health Animals Care and Use Committee, and were performed in accordance with an approved National Institute of Environmental Health Sciences animal study proposal.
All goat handling procedures were conducted in compliance with the guidelines on the Care and Use of Agricultural Animals in Agricultural Research and Teaching in France (Authorization no. 91-649 for the Principal Investigator, and national authorizations for all investigators. Approval from the Ethics Committee: 12/045)
Human fetuses (6-12 GW) were obtained from legally-induced normally-progressing terminations of pregnancy performed in Rennes University Hospital in France. Tissues were collected with women's written consent, in accordance with the legal procedure agreed by the National agency for biomedical research (authorization #PFS09-011; Agence de la Biomédecine) and the approval of the Local ethics committee of Rennes Hospital in France (advice # 11-48).

Note that full information on the approval of the study protocol must also be provided in the manuscript.

ChIP-seq

Data deposition

☒ Confirm that both raw and final processed data have been deposited in a public database such as GEO.

☒ Confirm that you have deposited or provided access to graph files (e.g. BED files) for the called peaks.

Data access links
May remain private before publication.

GSE128767; http://www.ncbi.nlm.nih.gov/geo/

Files in database submission

GSE128767_RUNX1_ChIP.peaks.bed.gz 189.7 Kb (ftp)(http) BED
GSE128767_fetalOvary_Input.bigWig 287.6 Mb (ftp)(http) BIGWIG
GSE128767_fetalOvary_RUNX1.bigWig 135.3 Mb (ftp)(http) BIGWIG

Genome browser session
(e.g. UCSC)

The ChIP-seq data are available in the ReproGenomics Viewer (https://rgv.genouest.org)

Methodology

Replicates

Two independent ChIP-seq experiments were performed using 20-30 µg of sheared chromatin from pooled embryonic ovaries (n=100-120 ovaries/ChIP)

Sequencing depth

ChIP-seq libraries were sequenced as single-end 75-mers by Illumina NextSeq 500
The total number of reads for RUNX1 Chip-seq for each biological replicate was 26,252,832 and 29,540,852 respectively.
The number of uniquely-mapped non-duplicate reads for each biological replicate was 8,932,674 and 15,036,698.

Antibodies

10 µl of homemade RUNX1 antibody was used (provided by Drs. Yoram Groner and Ditsa Levanon, the Weizmann Institute of Science, Israel, see reference #66).

Peak calling parameters

Binding regions were identified by peak calling using HOMER v.4.9 67 with FDR<1e-5. Called peaks were subsequently re-defined as 300mers centered on the called peak midpoints and filtered for a 4-fold enrichment over input and over local signal.

Data quality

ChIP-seq libraries were filtered to retain only reads with average base quality score >20. Reads were mapped against the mouse mm10 reference genome using Bowtie v1.2 with parameter "-m 1" to collect only uniquely-mapped hits. Duplicate mapped reads were removed using Picard tools MarkDuplicates.jar (v1.110).

Software

mapping: Bowtie v1.2 with parameter "-m 1" to collect only uniquely-mapped hits.
Duplicate mapped reads were removed using Picard tools MarkDuplicates.jar (v1.110).
binding regions were identified by peak calling using HOMER v.4.9 67 with FDR<1e-5.
