# Supplementary material for: RUNX1 maintains the identity of the fetal ovary through an interplay with FOXL2
Source: Nat Commun. 2019 Nov 11;10:5116. doi: 10.1038/s41467-019-13060-1 (PMC6848188; doi:10.1038/s41467-019-13060-1)
Supplement: Supplementary file 4 — Description of Additional Supplementary Files [file 41467_2019_13060_MOESM4_ESM.pdf]

Title: Supplementary Data 1:
Description: Probe lists associated with the Venn diagram from Figure 4g representing the genes differentially expressed in Runx1KO vs. control and for Foxl2KO vs. control ovaries at birth.

Title: Supplementary Data 2:
Description: Full Dataset for the microarray for control, Runx1 KO, Foxl2 KO, and Runx1/Foxl2 DKO newborn ovaries (n=4 / genotype). Tab2 allows the creation of a graph for a copied line from Tab1.

Title: Supplementary Data 3:
Description: Probe list for genes differentially expressed between Runx1/Foxl2 DKO vs. Ctr ovaries (Fold-Change >1.5). Tab1 represents the genes downregulated, Tab2 represents the genes upregulated.

Title: Supplementary Data 4:
Description: Gene lists for the Venn diagrams presented in Figure 7b (Tab1) and Figure 7c (Tab2).

Title: Supplementary Data 5:
Description: Probe list and fold-change values for the genes differentially expressed in Runx1/Foxl2 DKO vs. Foxl2 KO presented in Figure 7h. Tab1 represents the genes downregulated, Tab2 represents the genes upregulated.

Title: Supplementary Data 6:
Description: Full Dataset of RUNX1 ChIP-seq in fetal ovaries. Each row represents a significant peak for RUNX1 in fetal ovaries.

Title: Supplementary Data 7:
Description: Gene lists for pie chart from Figure 9a. The pie-chart identifies the genes differentially expressed in Runx1/Foxl2 DKO ovaries that are nearest to peaks for RUNX1 and/or FOXL2.
